# The O-specific polysaccharide lyase from the phage LKA1 tailspike reduces *Pseudomonas* virulence

**DOI:** 10.1038/s41598-017-16411-4

**Published:** 2017-11-24

**Authors:** Tomasz Olszak, Mikhail M. Shneider, Agnieszka Latka, Barbara Maciejewska, Christopher Browning, Lada V. Sycheva, Anneleen Cornelissen, Katarzyna Danis-Wlodarczyk, Sofya N. Senchenkova, Alexander S. Shashkov, Grzegorz Gula, Michal Arabski, Slawomir Wasik, Konstantin A. Miroshnikov, Rob Lavigne, Petr G. Leiman, Yuriy A. Knirel, Zuzanna Drulis-Kawa

**Affiliations:** 1 Institute of Genetics and Microbiology, University of Wroclaw, Wroclaw, 51-148 Poland; 2 Shemyakin-Ovchinnikov Institute of Bioorganic Chemistry, Russian Academy of Sciences, Moscow, 117997 Russia; 3 University of Texas Medical Branch, Department of Biochemistry and Molecular Biology, Sealy Center for Structural Biology and Molecular Biophysics, Galveston, TX 77555-0647 USA; 4 Vertex Pharmaceuticals (Europe) Ltd, Abingdon, Oxfordshire OX14 4RW UK; 5 Affinivax Inc., Cambridge, 02139-3543 Massachusetts, USA; 6 Laboratory of Gene Technology, KU Leuven, Leuven, 3001 Belgium; 7 N. D. Zelinsky Institute of Organic Chemistry, Russian Academy of Sciences, Moscow, 119991 Russia; 8 Department of Biochemistry and Genetics, Institute of Biology, The Jan Kochanowski University in Kielce, Kielce, 25-406 Poland; 9 Department of Molecular Physics, Institute of Physics, The Jan Kochanowski University in Kielce, Kielce, 25-406 Poland

## Abstract

*Pseudomonas* phage LKA1 of the subfamily *Autographivirinae* encodes a tailspike protein (LKA1gp49) which binds and cleaves B-band LPS (O-specific antigen, OSA) of *Pseudomonas aeruginosa* PAO1. The crystal structure of LKA1gp49 catalytic domain consists of a beta-helix, an insertion domain and a C-terminal discoidin-like domain. The putative substrate binding and processing site is located on the face of the beta-helix whereas the C-terminal domain is likely involved in carbohydrates binding. NMR spectroscopy and mass spectrometry analyses of degraded LPS (OSA) fragments show an O5 serotype-specific polysaccharide lyase specificity. LKA1gp49 reduces virulence in an *in vivo Galleria mellonella* infection model and sensitizes *P. aeruginosa* to serum complement activity. This enzyme causes biofilm degradation and does not affect the activity of ciprofloxacin and gentamicin. This is the first comprehensive report on LPS-degrading lyase derived from a *Pseudomonas* phage. Biological properties reveal a potential towards its applications in antimicrobial design and as a microbiological or biotechnological tool.

## Introduction

*Pseudomonas aeruginosa* has been marked as a critical pathogen, threatening society by its extensive antibiotic resistance emergence and abundant virulence mechanisms^1,2^. A key virulence factor of *P. aeruginosa* is its outer membrane composition, especially the smooth lipopolysaccharide (LPS) structure^3–7^. *P. aeruginosa* produces LPS with two forms of polysaccharides: A-band (common polysaccharide antigen, CPA) and B-band (O-specific antigen, OSA). Homopolymeric CPA is composed of trisaccharide repeating units of α-linked (α1-2, α1–3, α1–3) D-rhamnose residues. The heteropolymeric OSA includes diverse monosaccharides forming di- to pentasaccharide repeating units, and its structure provides the basis for serotype classification (O1 to O20)^8,9^. The polysaccharide chains of the B-band are longer than A-band (50 and 23 repeating units respectively), hereby masking the A-band polysaccharide and displaying a high immunogenicity^6–10^.

While all *P. aeruginosa* serotypes produce OSA, 14 out of 20 International Antigenic Typing Scheme (IATS) serotypes produce CPA correlating to isolates causing chronic lung infections^9–12^. Furthermore, the OSA length and distribution on the cellular surface provides resistance to cationic peptides, phagocytosis as well as bacterial elimination by human serum^12–15^. Indeed, *P. aeruginosa* mutants with rough LPS (especially lacking in B-band elements) are sensitive to human serum, whereas smooth LPS determines resistance to its killing effect^6,16,17^. In addition, anti-OSA antibodies were protective against *Pseudomonas* infection in mice, whereas anti-CPA antibodies were not^6,18^. This makes the B-band LPS an excellent target for new antimicrobials. On the other hand, during the establishment of mucoid *P. aeruginosa* in the Cystic Fibrosis (CF) patient’s lungs, phenotypic LPS changes occur, which result in a decreased level of OSA production^6,17,19–22^.

With the dramatic increase of antibiotic resistance in *P. aeruginosa*, alternatives like bacterial viruses or bacteriophages are currently being re-evaluated^23^. Phage therapy was used in the first half of the twentieth century, but was largely abandoned when antibiotics were discovered and developed as pharmaceuticals in WWII^24^. Also, the development of molecular biology techniques has enabled the evolution of „traditional” phage therapy towards new phage-inspired antibacterials, which includes the use of phage-encoded proteins responsible for bacterial envelopes^25–27^.

 Bacteriophage-encoded depolymerases are highly specific enzymes, acting at the first step of phage infection and serve for specific recognition of host cell receptor, as well as for the degradation of polysaccharides^27^. The polysaccharide-degrading enzymes are primarily virion-associated proteins like tail fibers, tailspikes, base plate or neck, whereas some are soluble proteins, released during bacterial lysis^27,28^. These enzymes target structural polysaccharides (LPS, capsular polysaccharides, peptidoglycan) and essential components of the biofilm matrix^27–30^. As such, phage depolymerases have been suggested as potential anti-virulence agents, effective in fighting and prevention bacterial infections, and as compounds to degrade bacterial biofilms^27–31^. In addition, depolymerases have also been proposed as antibiotic adjuvants and as diagnostic tools^31^. However, the structural and biochemical information on these enzymes remains limited to a number of type phages infecting enteric bacteria. These include rhamnosidases encoded by some *Salmonella* phages (P22, SP6, 9NA, Det7, P27, KB1, epsilon15) and *Shigella* phage Sf6, and coliphages HK620 (endo-N-acetylglucosaminidase) and Ω8 (endo-α-1,3-mannosidase)^30^. We here report a comprehensive microbiological, biochemical and structural analysis of the *Pseudomonas* phage LKA1 tailspike-associated depolymerase, the first (recombinant) LPS-degrading enzyme specific for *P. aeruginosa*.

## Results and Discussion

### *Pseudomonas* phage LKA1 encodes a tailspike with depolymerase activity

*Pseudomonas* phage LKA1 is an estranged member of the *Phikmvvirus* genus within the *Autographivirinae*, and forms an expanding halo upon prolonged incubation in which bacteria survive. An infectivity screen on available LPS-mutants indicates that LKA1 infection requires the B-band O-polysaccharide of LPS (OSA) (Table 1), similarly to siphovirus D3, yet contrary to most *P. aeruginosa*-infecting *Phikmvvirus* phages which are type IV pili dependent^32^. Phage LKA1 encodes a trimeric tailspike protein (LKAgp49) with a typical conserved N-terminal structure-associated domain and an unconserved central and C-terminal region with predicted polysaccharide binding/degrading activity (Pfam 12708 and right-handed β-helices)^33–37^. Spotting of recombinant LKA1gp49 on LPS-mutants lawn indeed results in the characteristic halo zone, which supports that enzyme activity is based on the degradation of B-band O-polysaccharide of LPS (Table 1).

**Table 1:** The specificity identification on outer membrane mutants with the *Phikmvvirus* and LKA1gp49 recombinant protein. (L = lysis, H = Halo/opaque zone formation).

	Strains	LKA1	Other *Phikmvvirus*	LKA1gp49
Wild type	PAO1_k_	L	L	H
Wild type without pili	PAO1_p_	L	/	H
Flagella and pili mutants	^a^wt *fliC*/wt *pilA*	L	L	H
	Δ *fliC*/wt *pilA*	L	L	H
	wt *fliC*/Δ *pilA*	L	/	H
LPS-mutants	^*b*^Δ *rmd* (A^−^ B^+^)	L	L	H
	^c^Δ *waaL* (A^−^ B^−^)	/	L	/
	^d^Δ *rmlC* (A^−^ B^−^ Core^-^)	/	/	/
	^e^Δ *wbpL* (A^−^ B^−^)	/	L (except LKD16, LUZ2)	/
Derived from a	^f^Δ *fli*C/wt *algC*	LH	H	H
Wildtype without pili	Δ *fli*C/Δ *algC*	/	/	/

^a^knock out mutants of *fliC* and *pilA* don’t display functional flagella or pili, respectively.

^b^ *rmd* (GDP-4-keto-6-deoxy-D-mannose reductase) knock out mutants are deficient in A-band LPS synthesis, but produce smooth B-band LPS.

^c^ *waaL* encodes the ligase for both the A- and B-band O-polysaccharide.

^d^ *rmlC* mutants display a truncated outer core lacking the 1,3- or 1,6-linked rhamnose residue and display no A- or B-band O-polysaccharide.

^e^ *wbpL* encodes the initial glycosyltransferase which is necessary for initiation of both A- and B-band LPS synthesis.

^f^ *algC* (phosphomannose mutase and phosphoglucose mutase) mutants are deficient in alginate synthesis, in the A- and B-band O-polysaccharide and display a truncated outer core.

### Characterization of enzyme in solution

The activity of recombinant LKA1gp49 was evaluated in serial dilution giving a visible opaque zone at the lowest concentration of 0.5 µg/ml (pH 7.4; RT) later considered as 100% (Supplementary Table 1, Fig. S1A). This enzyme is completely inactivated after 1 hour at pH 2, while the protein loses about 90% of its activity at pH 4, but still creates a visible halo zone at a concentration of 5 µg/ml. The enzyme remains stable for at least three hours at pH values between 8 and 10. One hour of incubation at pH 6 or 12 reduces the activity of LKA1gp49 by approximately 50% (Supplementary Table 1). The activity optimum under slightly alkaline conditions has also been observed for *A. vinelandii* phage lyase and coliphage KflA lyase, but differs from polysaccharide specific depolymerase, which stays active in slightly acidic conditions^38,39^. Interestingly, the protein remains fully active (100%) after one hour of incubation at 60 °C–80 °C, as well as at 90 °C for 15 min. Sixty minutes of incubation at 90 °C results in a 50% decrease of activity (Supplementary Table 1). Monomers have only been observed upon heating (5 min, 95 °C) (Fig. S1B). This enzyme is thermostable since at least 15 min of incubation at 100 °C is required to inactivate 90% of the protein, contrary to other depolymerases defined as thermostable, which lose the activity after 10 min of incubation at 68 °C or 30 min at 70 °C^40,41^.

### Crystal structure of LKA1gp49

**Figure 1:**
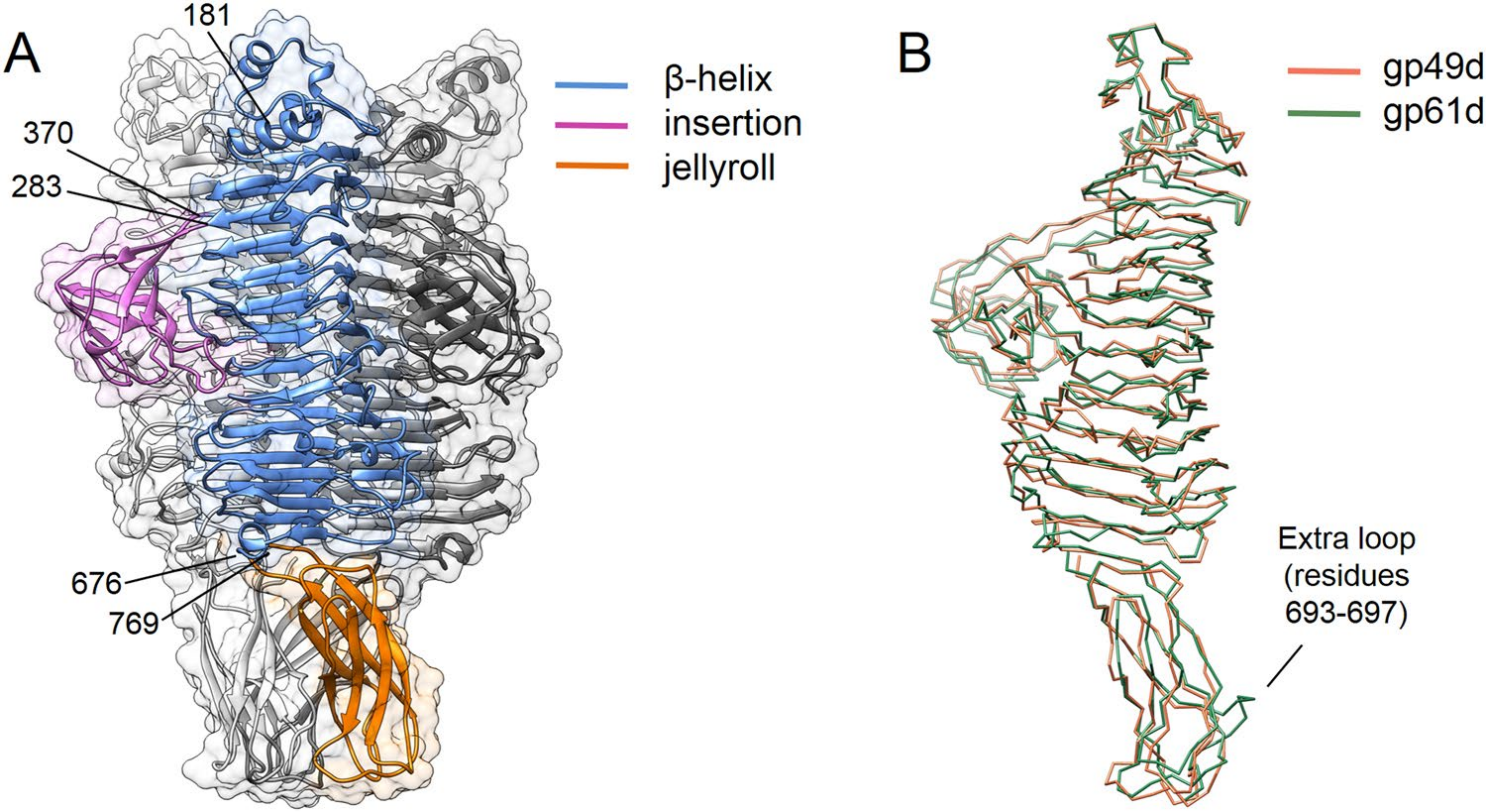
Structure of gp49d. (**A**) Ribbon diagram of the gp49 trimer with each domain labeled in a distinct color within the same monomer. The other two monomers are colored in two different shades of gray. Residue numbers at domain borders are indicated. The semi-transparent molecular surface is also shown. (**B**) Superposition of gp49d and gp61d. The small extra loop in gp61d is labeled.

For crystallization, the N-terminal particle-binding domain of LKAgp49 (residues 1–167) was removed. The resulting mutant – gp49d – constitutes a ‘catalytic module’ of the tailspike. Initial extensive crystallization trials failed to produce single isotropically diffracting crystals. Therefore, it was decided to first solve the structure of a homologous protein by a *de novo* phasing technique (SAD, MAD, etc.) and subsequently solve the structure of gp49d by molecular replacement (deposited to the PDB database as structure 4RU4). An equivalent fragment of gp61 of phage phi297 (residues 1–142, named gp61d) was cloned, expressed, purified and crystallized. The crystals diffracted to 1.5 Å resolution and the crystal structure was solved by the SeMet SAD technique (PDB: 4RU5). Eventually, one crystal of gp49d gave a clean and isotropic diffraction pattern that extended to 1.9 Å resolution. As expected, the structure of gp49d could be easily solved by molecular replacement with phi297 gp61d as a search model. Gp49d and gp61d display 32% amino acid sequence identity and can be superimposed with a root mean squared deviation of 1.6 Å between 572 (or 97%) equivalent C-alpha atoms (Fig. 1). The main chain traces of the two proteins are virtually identical (Fig. 1) except for a small extra loop in the C-terminal domain of gp61 (residues 693–697). Several N-terminal residues are disordered in both structures: 168–180 in gp49d and 142–146 in gp61d. Gp49d can be divided into three domains (Fig. 1): (1) a β-helix typical to many tailspikes (residues 181–283 and 371–675); (2) a β-barrel or an insertion domain that is positioned on a side of the β-helix and that interrupts the run of polypeptide chain comprising the β-helix (residues 284–370); and (3) a β-sandwich or jellyroll C-terminal domain (residues 676–769). For each domain, a search for similar structures in the PDB database was performed with the help of the DALI webserver^42^.

**Figure 2:**
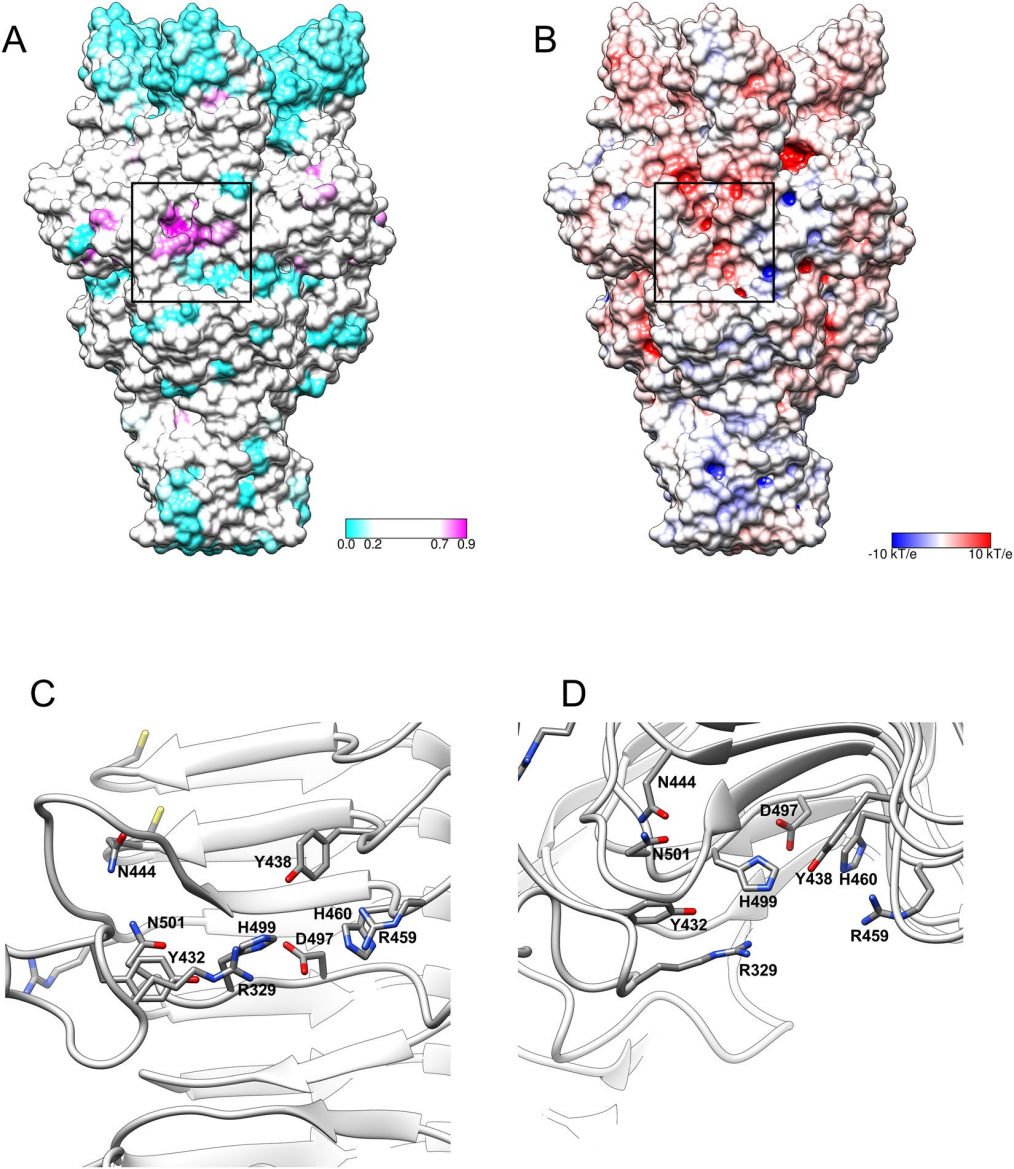
Putative active site of gp49d. (**A**) and (**B**) Amino acid sequence conservation and electrostatic potential are mapped onto the molecular surface of gp49d. The color scale bar for each panel is given. The black rectangles indicate the area shown in panels (**C**) and (**D**). (**C**) and (**D**) Two roughly orthogonal views of the most conserved residues that are likely responsible for creating the active site.

The β-helix of gp49d is similar to other β-helices that bind and cleave oligo- and polysaccharide substrates^43^. However, the actual mode of substrate binding, the location of the substrate-binding/active site on the β-helix and the enzymatic mechanisms vary in different β-helices^44^. None of the proteins identified by DALI as being structurally similar to gp49d displays an active site that is composed of amino acids that perfectly match residues of gp49d. The active site of an enzyme is its most conserved part, and NMR analysis (described below) shows that gp49d is a lyase. We, therefore, mapped amino acid sequence conservation onto the molecular surface of gp49d. This procedure identifies a region of high conservation roughly in the middle of the β-helix (Fig. 2). This region is also in the middle of a negatively charged groove that runs through the length of the β-helix. The most conserved residues form an intricate system connected by hydrogen and ionic bonds with Asp497 coordinating His494 and His499 (Fig. 2). Asp497 also forms a hydrogen bond with the main chain N atom of Thr464. The three residues are likely to be the most important in the catalysis with Asp497 being the critical residue. The smallest soluble fragment of gp49 that could be recombinantly expressed and was enzymatically active comprised residues 389–584 that constitute a large part of the β-helix (Fig. 1).

The run of the β-helix is interrupted by a six-stranded β-barrel insertion domain (residues 283–370, Fig. 1). It makes the substrate-binding groove of the β-helix deeper and participates in the creation of the active site. A highly conserved Arg329 extends towards the active site where it forms a hydrogen bond with His499 (Fig. 2). Conserved Arg459 emanates from the opposite side of the β-helix’s groove towards these two residues and together with Arg329 forms a constriction in the groove. Thus, this insertion domain may participate in substrate binding and processing. The role of this domain in the *in vitro* enzymatic activity is unclear, but this domain might be important for the function of the protein when it is part of the phage particle. Interestingly, DALI shows that a domain with a similar structure is found in the major capsid protein of phage P22 (residues 222–345) where it likely functions to stabilize the interactions between the subunits^45^, and in the V-type ATPase (residues 1–75) where its function is unknown^46^.

The structure of the C-terminal jellyroll β-sandwich domain of gp49 is most similar to the discoidin-1/2 domains. Discoidins bind galactose and N-acetylgalactosamine^47^, although the role of this jellyroll fold domain in oligosaccharide binding has not been demonstrated. A number of phage tailspike and tail fiber proteins carry a similar domain at their C-termini (e.g. phages AP22 and SF6)^35^. Besides a similar domain is found in the *Helix pomatia* agglutinin (N-acetylgalactosamine (GalNAc) binding lectin)^48^, in the FlhE protein (possible flagellum assembly chaperone)^49^, and even in the hyperthermostable subtilisin-like serine protease Tk-SP from *Thermococcus kodakaraensis* where it appears to play a role in an increased stability of the protein^50^. Summarizing all the findings, these domains are likely to have evolved to bind to oligo- and polysaccharide and, possibly, stabilize the trimeric structure of tailspikes.

### LKA1gp49 is an O-specific polysaccharide lyase

**Figure 3:**
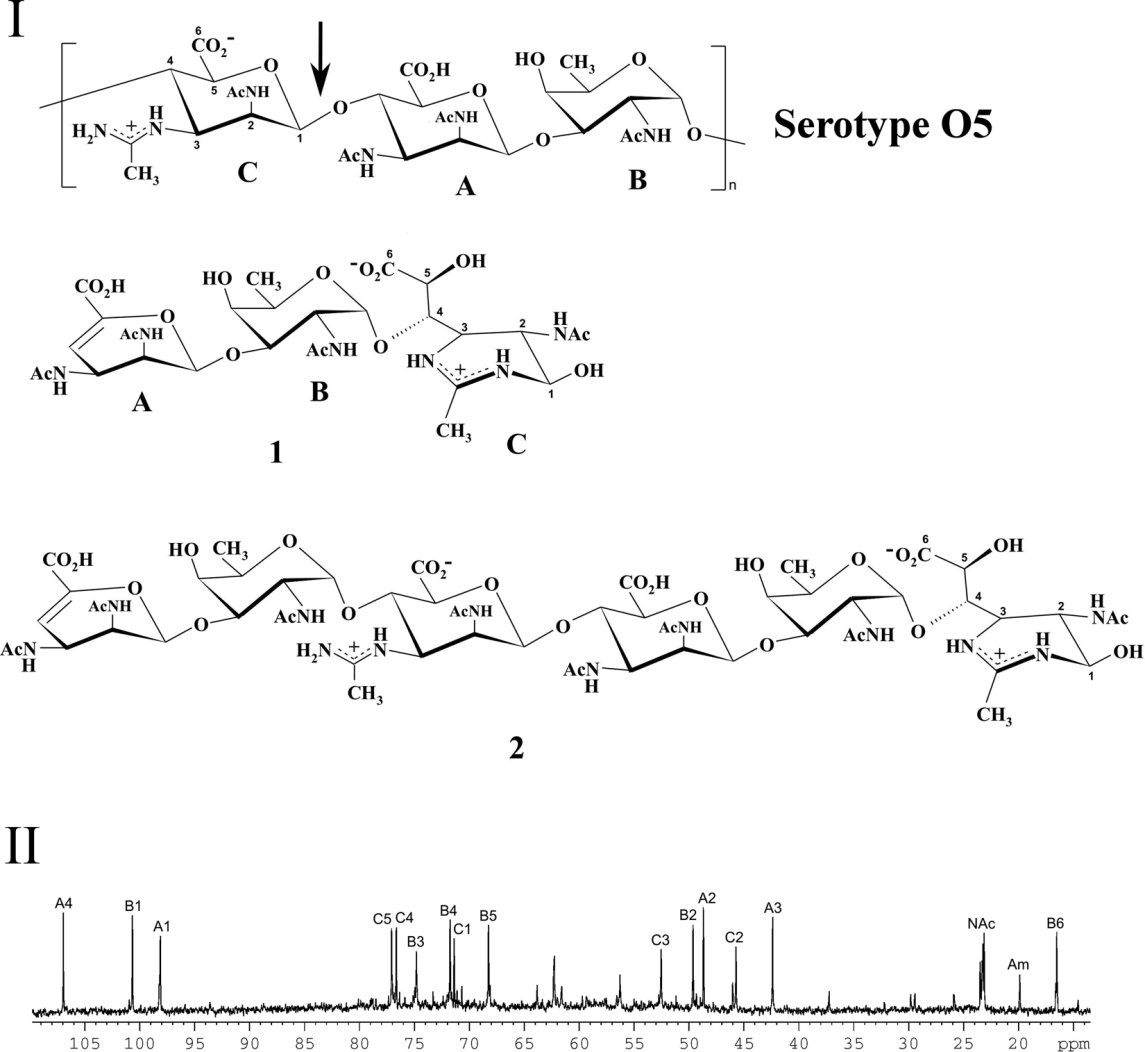
Structure and NMR spectrum of *P. aeruginosa* O5 oligosaccharide. **I**. Structures of the OSA of *P. aeruginosa* O5 and phage degradation products **1** (major) and **2** (minor). Arrow indicates the point of cleavage in the OSA. **II**.^13^С NMR spectrum of oligosaccharide **1**. Numbers refer to carbons in sugar residues denoted by letters as indicated in Supplementary Table 1 and Fig. 3 II. Am indicates Me of the acetamidino group in residue C.

The structure of the O-specific antigen (OSA) of the lipopolysaccharide of *P. aeruginosa* IATS serotype O5 was first reported for strain 170005^51–53^. The OSA has a trisaccharide repeat (O-unit) consisting of one residue each of 2-acetamido-2,6-dideoxy-d-galactose (d-FucNAc), 2,3-diacetamido-2,3-dideoxy-d-mannuronic acid, and 2-acetamido-3-acetimidoylamino-2,3-dideoxy-d-mannuronic acid (Fig. 3I).

The OSA was isolated from the lipopolysaccharide by mild acid hydrolysis and cleaved with the recombinant full length LKA1gp49. The products were fractionated by gel-permeation chromatography on TSK HW-40 to give a major lower-molecular mass oligosaccharide (**1**) and a minor higher-molecular mass oligosaccharide (**2**). Structures of both compounds were established by nuclear magnetic resonance (NMR) spectroscopy and high-resolution electrospray ionization mass spectrometry (HR ESI MS). The ^1^H NMR and ^13^C NMR (Fig. 3II) spectra of **1** were assigned using two-dimensional ^1^H, ^1^H correlation spectroscopy (COSY), ^1^H, ^1^H total correlation spectroscopy (TOCSY), ^1^H, ^13^C heteronuclear single-quantum coherence (HSQC), and ^1^H, ^13^C heteronuclear multiple-bond correlation (HMBC) experiments (Supplementary Table 2). Spin systems for three sugar residues were revealed, which were designated residues **A**–**C** according to their sequence in the trisaccharide (Fig. 3I). Residue **B** was identified as α-Fuc*p*NAс by (i) the presence of a CH_3_ group at position 6 (δ_H_ 1.19, δ_С_ 16.5), (ii) the presence of an AcNH group at position 2 (δ_С_ 49.6), and (iii) coupling constants specific for the α-*galacto* configuration (Supplementary Table 2).

Structure of residue **A** as a 4,5-unsaturated 2,3-diacetamido-2,3-dideoxyhexuronic acid was established by (i) the presence of AcNH groups at positions 2 and 3 (δ_С_ 48.7 and 42.4, respectively), (ii) low-field positions of the signals for C4 and C5 at δ_С_ 107.0 and 145.3, respectively, (iii) the lack of H5 proton, and (iv) a correlation between H4 and a CO_2_H group (C6) at δ_H_/δ_С_ 5.92/169.7 in the ^1^H,^13^C HMBC spectrum. Structure of residue **C** was inferred from (i) the presence of acylamino groups at positions 2 and 3 (δ_С_ 45.7 and 52.5, respectively), one of which is an AcNH group (δ_H_ ~2, δ_C_ ~23 and ~175) and the other an acetamidoylamino (Am) group (δ_H_ 2.35, δ_C_ 19.9 and 163.4), and (ii) correlations of H5 with a CO_2_H group (C6) at δ_H_/δ_С_ 4.52/177.5 and H1 with C2 of Am at δ_H_/δ_С_ 5.04/163.4 in the ^1^H,^13^C HMBC spectrum. That the pyranose ring is open in residue **C** followed from a relatively high-field position of the C1 signal at δ_С_ 71.4 and the lack of correlation between H1 and C5 in the ^1^H,^13^C HMBC spectrum typical of pyranosides.

The linkage and sequence analyses of **1** was performed using a two-dimensional ^1^H,^1^H rotating-frame nuclear Overhauser effect spectroscopy (ROESY) experiment, which revealed **A** H1/**B** H3 and **B** H1/**C** H4 correlations at δ_H_/δ_H_ 5.41/4.16 and 4.82/4.06, respectively. These data were confirmed by the ^1^H,^13^C HMBC spectrum, which showed **A** H1/**B** C3 and **B** С1/**C** H4 correlations at δ_H_/δ_C_ 5.41/74.8 and δ_С_/δ_H_ 100.7/4.06, respectively. The structure of **1** shown in Fig. 3I was confirmed by the negative ion HR ESI mass spectrum, which showed a [M-H]^−^ ion peak at *m/z* 701.2645 for a compound with the molecular formula C_28_H_42_N_6_O_15_ (calculated ion mass 701.2635 Da). Mass spectrum of **2** showed a peak for a [M-H]^−^ ion at *m/z* 1403.5362 indicating that this compound is a hexasaccharide having the structure shown in Fig. 3I (molecular formula C_56_H_84_N_12_O_30_, calculated ion mass 1403.5344 Da). The structure of **2** was confirmed by NMR spectroscopy as described above for compound **1** (data not shown).

A comparison of the structures of trisaccharide **1** and the O-unit (Fig. 3I) showed that the OSA was cleaved by the linkage between the residues **C** and **A** by the mechanism of β-elimination. The cleavage was accompanied by a rearrangement in residue **C** to afford a tetrahydropyrimidine ring involving the acetimidoylamino group. Based on these data, it was concluded that the tailspike protein LKA1gp49 is a specific polysaccharide lyase.

In terms of enzymatic specificity, the phage depolymerases can be classified into two main groups: (i) O-glycosyl hydrolases (EC 3.2.1) cleaving specifically the O-glycosidic bonds of a polysaccharide using a water molecule, and (ii) lyases (EC 4) cleaving a glycosidic bond by β-elimination in an uronic acid with the concomitant introduction of a double bond. The latter enzymes are mostly found as viral-associated depolymerases degrading bacterial capsular polysaccharides or possessing hyaluronate, pectate/pectin and alginate lyase activity^30,54^. Experimental data characterizing OSA depolymerases was derived from phages propagating on *Salmonella* and *Shigella* host (rhamnosidases cleaving the α-1,3 O-glycosidic bonds; deacetylase) and *E. coli* (endo-N-acetylglucosaminidase and endo-α-1,3-mannosidase)^30^. No phage lyase degrading a *P. aeruginosa* LPS polysaccharide has been reported so far.

### Activity of LKA1 depolymerase in carbohydrate-mediated degradation

First, the carbohydrate-mediated degradation by the LKA1gp49 depolymerase was verified on isolated PAO1 LPS and on the biofilm matrix, using a previously established interferometry method which optically measures quantitative changes in biofilm matrix permeability for low molecular compounds^55,56^. First, the LPS digestion activity of 50 µg/ml depolymerase was evaluated on the PAO1 host strain and its *rmd* knock out mutant (deficient in CPA) producing only smooth B-band LPS (Fig. S2). The SDS-PAGE analysis (Fig. S2) shows that the enzyme is able to truncate almost completely the OSA chain of the B-type within one hour treatment. The optimal concentration of recombinant enzyme effectively degrading the LPS within one hour can now be used in experiments revealing the anti-virulence properties (efficiency of phagocytosis, serum complement resistance of depolymerase-treated PAO1 culture, in synergy with antibiotics and reduction of pathogenicity of PAO1 strain infecting *G. mellonella* larvae).

**Figure 4:**
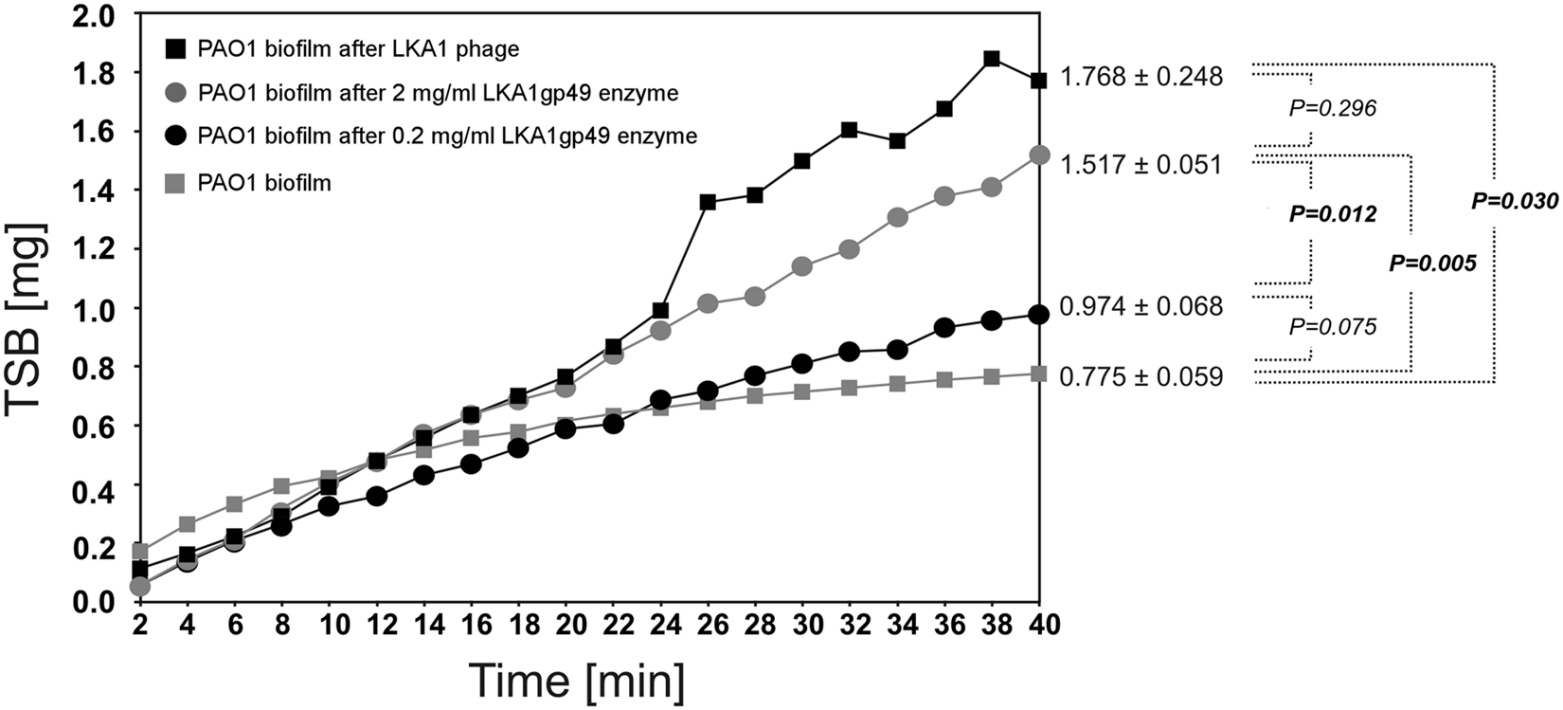
Laser interferometry analysis of TSB medium diffusion through PAO1 biofilm treated with LKA1 phage (5 × 10^8^ pfu/ml) or LKA1gp49 enzyme (2 and 0.02 mg/ml). Untreated biofilm was used as a control. The results are presented as the means ± SD from three independent experiments.

Second, the biofilm disruption effect was evaluated using laser interferometry by adding phage LKA1 and recombinant LKA1gp49 to the hydrophylic nephrophane on a 72h-grown biofilm and measuring the reduction in membrane coverage^57^. The PAO1 biofilm was treated with virulent LKA1 phage particles (5 × 10^8^ pfu/ml) and with two concentrations of recombinant depolymerase for 4 h at 37 °C. The amount of TSB medium transported in 40 min through the biofilm after treating with enzyme at the concentration of 0.02 mg/ml did not differ significantly from untreated biofilm-covered membrane (0.974 mg and 0.775 mg, respectively) (Fig. 4).

After application of phage LKA1 (5 × 10^8^ pfu/ml) a 1.5-fold increase of diffusion rate (1.768 mg after 40 min; *P* = 0.03) was observed. Similar increase was observed for biofilm treated with 2 mg/ml LKA1gp49 depolymerase (1.517 mg after 40 min; *P* = 0.005). The high concentration of recombinant enzyme digesting OSA of LPS was efficient in releasing the matrix exopolysaccharides, enhancing the diffusion trough the biofilm structure. The higher enzyme concentration enzyme was necessary as the diffusion of protein through the PAO1 biofilm might be limited by its size or physicochemical properties. Moreover, these results may suggest that LPS molecules are strongly involved in biofilm stability or that the exopolysaccharides building the matrix are chemically identical to *Pseudomonas* OSA. As such, Chen and Zhu^58^ describe the production of rhamnolipids by *Pseudomonas* cells embedded in the biofilm structure. Rhamnolipids, as biosurfactants, stimulate LPS production and increase the release of outer membrane vesicles (OMVs) to the medium (biofilm matrix). It has already been shown that OMVs constitute an important component of *P. aeruginosa* biofilm matrix. In addition, the CPA expression is responsible for biofilm development and the properties of sessile cells embedded in biofilm structure^59^. Although, the CPA and OSA expression do not influence OMV formation and their release profile, both affect the size and protein content of OMVs produced by *Pseudomonas* cells. Based on this information, we may conclude that LPS-degrading depolymerases derived from a phage might be successfully used as an agent to disrupt the matrix, which could further allow other antibacterial components to penetrate the biofilm structure.

### Depolymerase treatment of PAO1 does not influence the efficiency uptake by phagocytic cells

The impact of LKA1gp49 on *P. aeruginosa* virulence can be measured by the recognition and engulfment rate by macrophages, examined using THP-1 cell line. Results of these experiments show that in both cases (PAO1 treated with LKA1gp49 and non-treated control) about 5.5 × 10^6^ bacterial cells adhered to the macrophages surface receptors and approximately 6.0 × 10^4^ have been taken up. In this particular experimental system, the truncation of the chain-specific LPS did not directly affect the intensity of phagocytosis by macrophages, although theoretically the virulence of the strains exposed to the enzyme should be reduced. It has been shown, both *in vitro* and *in vivo*, that the efficient phagocytosis of *P. aeruginosa* cells is mostly conditioned and initiated by the presence of bacterial flagella^60,61^. The activation of PI3K/Akt pathway by flagellar swimming motility resulted in the increased engulfment by phagocytic cells such as macrophages, neutrophils, and dendritic cells, as well as in TLR-based activation^62^.

### Depolymerase treatment of PAO1 increases the susceptibility to complement-mediated serum killing

**Figure 5:**
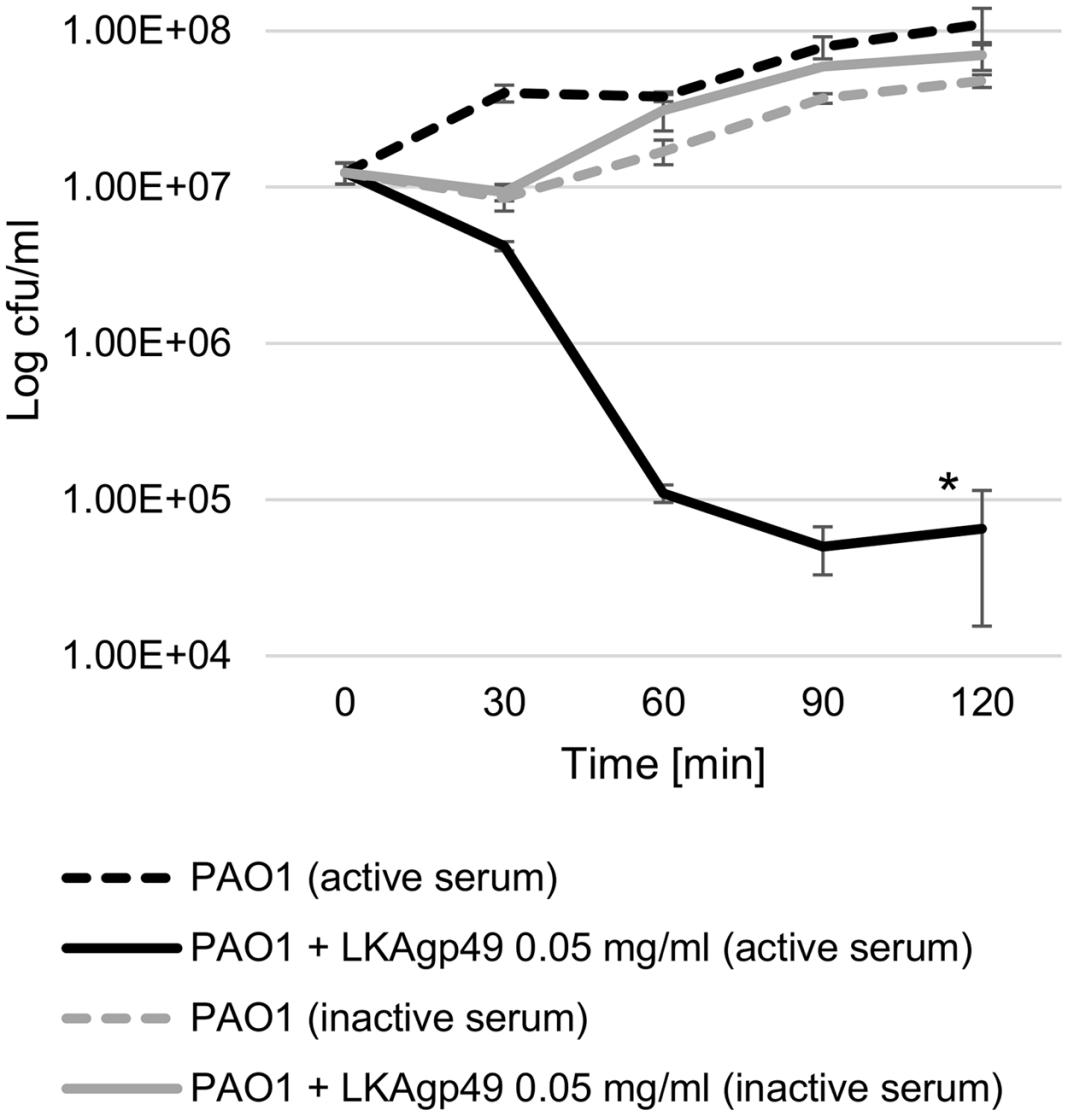
The lytic activity of serum complement tested on PAO1 culture incubated with or without LKA1gp49 (0.05 mg/ml) in the presence of 50% active and heat-inactivated sheep serum.

The complement system, composed of distinct plasma proteins, is a part of the innate immune response. It plays an important role in pathogen opsonization and in the induction of inflammatory responses. There are three major pathways of complement activation leading to: (i) the binding of activated complement proteins to bacterial cell accelerates the phagocytosis process; (ii) the recruitment and activation of phagocytes; and (iii) the formation of membrane-attack complex (MAC) creating pores in the bacterial cell leading to its lysis^63^. In this experiment, the influence of LKA1gp49 depolymerase on PAO1 serum resistance is examined (Fig. 5). Figure 5 clearly indicates that untreated PAO1 cells are not susceptible to complement lytic activity, whereas the depolymerase-truncated LPS loses its protective properties and the MAC complex is able to form pores in the PAO1 membrane leading to bacterial cell lysis. The active serum causes a 4 log colony count reduction after 90 min of incubation, while inactive serum does not possess any adverse effect on the enzyme-treated and -untreated culture. This experiment shows that OSA chains are the only elements responsible for complement resistance, since other PAO1 virulence agents should remain unchanged, after LPS-targeted depolymerase treatment. This experiment suggests that phage-borne depolymerases exhibiting high specificity to particular virulence agents (LPS, capsule, biofilm matrix), could be considered a precise tool to study the biological governance of each virulence element independently. This is contrary to commonly-used knock-out mutants used to determine the characteristic features of different virulence agents. Moreover, the depolymerase can be used as a specific tool to facilitate the generation of bacterial deficient mutants without changing the genetic background.

### Depolymerase application increases the survival rate of PAO1 infected wax moth larvae

**Figure 6:**
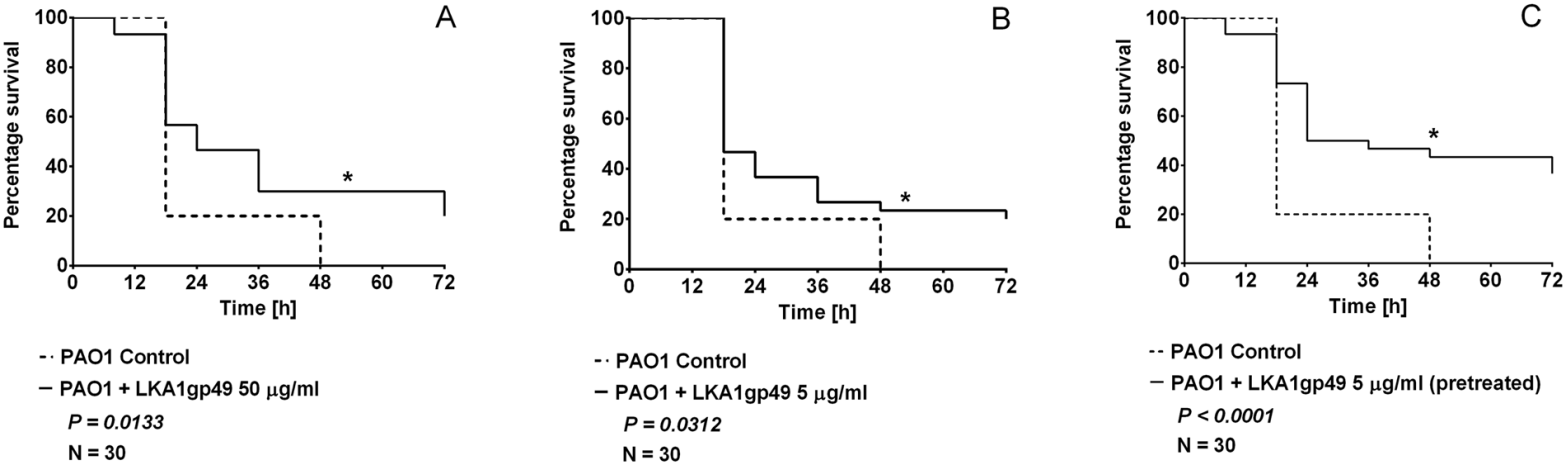
The influence of recombinant LKA1gp49 depolymerase on PAO1 virulence tested in *Galleria mellonella* infection model. (**A**) Larvae treatment with 50 µg/ml LKA1gp49 depolymerase; (**B**) Larvae treatment with 5 µg/ml LKA1gp49 depolymerase; (**C**) Larvae infected with PAO1 cells pretreated with 5 µg/ml LKA1gp49 depolymerase for 1 hour. The control consisted of infected larvae without therapy. Statistical analysis was calculated for pair wise comparisons between infected larvae and phage treated infected larvae using Mantel-Cox test (denoted *P*-values < 0.05).

Indeed, to evaluate the effect of B-band LPS truncation on PAO1 pathogenicity, LKA1gp49 depolymerase-treated *P. aeruginosa* cells were evaluated in the *Galleria mellonella* model. The experiments were performed in two setups (‘curative’ & ‘prophylactic’). Both experimental setups reveal that treatment with LKA1gp49 results in a significant prolongation of the larvae lifespan (Fig. 6). After 24 hours of treatment, the larvae survival rate is at least 20% higher compared to the control (i.e. infected larvae without enzyme application) (Fig. 6A–C). In the case of depolymerase administration after PAO1 injection (Fig. 6A,B), similar results are obtained, independent of the LKA1gp49 concentration (5 or 50 µg/ml) used. The application of LKA1gp49 depolymerase extends the survival of about 20% caterpillars up to 72 hours, compared to control larvae dying 48 h post injection. In the second experimental setup (Fig. 6C) the PAO1 culture was incubated for one hour with LKA1gp49 (5 µg/ml) before injection, resulting in an even greater survival rate of tested larvae. After 24 hours of incubation, 50% of the larvae remain alive (about 30% more than in the control). Moreover, 35% of the insects survive to the end of the experiment (more than 72 hours). These results prove that the OSA-degrading depolymerase is an efficient anti-virulence agent, able to reduce *Pseudomonas* propagation in this *in vivo* model. However, conclusions based on this model need to be interpreted with caution. *G. mellonella*, as a representative of the *Lepidoptera* order, has a quite rudimentary immune system, based on the innate mechanisms for combating pathogens in the humoral and cellular way^64^. During the infection of a highly entomopathogenic *P. aeruginosa*, many virulence factors, including metalloproteinases, are secreted outside of the cell. These proteases stimulate the immune system but in an advanced stage of infection cause insect immune components breakdown^65^. However, all features of insect immunity and PAO1 virulence agents remain unchanged in this experimental setup. The only variable in the experimental procedure is the bacterial LPS, where B-type O-specific chain has been shortened by the action of LKA1gp49 depolymerase. Although the pathogenesis of *P. aeruginosa* in *G. mellonella* largely depends on other virulence factors (proteolytic enzymes), the LPS O-specific chain turns out to be also important for *Pseudomonas* pathogenicity (smooth strains are more virulent), consistent with historical observations^66^.

### The antimicrobial activity of LKA1gp49 in synergy with antibiotics

The data described above indicate the phage-borne depolymerase may be a potentially useful anti-virulence agent, hence it is important to determine the possible application of enzyme together with antibiotics in a combined therapy. Thus, the interesting issue is to find out if the enzyme is undergoing any positive or adverse interactions with antibacterial drugs. Therefore, the recombinant LKA1gp49 depolymerase was tested in combination with anti-pseudomonal antibiotics commonly used in therapy, to determine the effect of LPS B-band degradation on drugs efficacy. The 50 µg/ml enzyme concentration was used, established as efficient in the LPS degradation assay. The MICs for both antibiotics were evaluated by the broth dilution method as recommended by the CLSI committee (http://clsi.org/). The MICs for ciprofloxacin (CIP) and gentamicin (GE) were found to be 0.125 µg/ml and 0.5 µg/ml, respectively. The addition of depolymerase improves CIP efficiency reducing the OD_600_ from 1.2 ± 0.05 to 0.5 ± 0.01, at ½ MIC and decreasing the colony count from 1.2 × 10^9^ cfu/ml to 5 × 10^7^ cfu/ml. The reduction of cfu/ml for GE ranges around one log at the ½ MIC drug concentration. In general, the MICs for both drugs does not change in the presence of OSA depolymerizing enzyme. Thus, consistently with previous report for a *Klebsiella* phage-derived depolymerase^39^, the *in vitro* experiments shows that LKA1gp49 does not potentiate the efficacy of GE or CIP in PAO1 strain as a model. Moreover, the idea to combine standard antibiotics with phage-borne enzyme degrading OSA chain might lead to undesirable reduction of drug efficiency, e.g. for positively charged aminoglycosides which require the ionic interactions with bacterial cell surface to be actively transported through the membrane^67^. These drugs displace divalent cations that crosslink and stabilize LPS chains. Thus, the most common pseudomonas resistance mechanism is based on the truncation of smooth LPS to rough LPS phenotypes^68^. In the case of the LKA1gp49 degrading the OSA, we expected a reduced activity of GE/depolymerase combination. However, the presence of remaining CPA is sufficient to maintain divalent cations on bacterial surface and the final antibacterial activity of GE is not impacted. The second anti-pseudomonal broad spectrum antibiotic tested is CIP (fluoroquinolone). This amphoteric molecule with two potential ionizable groups facilitates its interaction with lipids embedded in bacterial membrane structure. CIP forms four different microspecies (neutral, zwitterion, positively and negatively charged) depending on the pH of the solution which properties condition the efficient transport of antibiotic molecules through bacterial envelopes and cytoplasmic membranes^69^. The addition of LKA1gp49 LPS depolymerase may slightly increase the permeability of LPS layer which resulted in the reduction of the colony count in CIP treated PAO1 culture.

## Conclusions

As indicated by infection-analysis on outer membrane mutants, *Pseudomonas* phage LKA1 requires the LPS B-band OSA as primary receptor and displays characteristic opaque-looking halo zones. Phage LKA1 possesses a virion-associated polysaccharide-degrading enzyme as a trimeric tailspike protein (LKA1gp49). Its crystal structure reveals an interesting three-domain structure and allowed us to pinpoint the putative active site region of this enzyme. LKA1gp49 is a lyase that degrades the O5-serotype specific polysaccharide. This enzyme degrades LPS molecules embedded in the cell envelope and disperses the biofilm matrix, resulting in an increased diffusion rate for small molecules. LKA1gp49 lyase efficiently reduces *P. aeruginosa* virulence in the *in vivo Galleria mellonella* infection model, and sensitizes bacterial cells to the lytic activity of serum complement. LKA1gp49 could also be a potential additive for antimicrobials as it does not interrupt the efficacy of ciprofloxacin and gentamicin. Moreover, owing to its high specificity OSA-degrading enzyme could be used for microbiological/biotechnological applications.

## Methods

### LKA1gp49 depolymerase recombinant expression and purification

Purified genomic DNA of *P. aeruginosa* phage LKA1^70^ served as a template for amplification of chosen ORF in a PCR reaction using following primers (LKA1gp49F: ATGGCGCAAACACCCAG, LKA1gp49R: TCACACCTCATAAATACCTG and LKA1gp49_389-584 F: ATGGGACTGTTAGTGGAGGCTG, LKA1gp49_389-584 R: TTACGTTGTATTTGTAGTGAGGAC). The PCR products were cloned into commercially available pEXP5-NT/TOPOR (Invitrogen, Thermo Fisher Scientific, Waltham, MA, USA) expression vector according to previously described methodology^39^. The large scale purification of recombinant protein was performed on a 1 ml HisTrap HP column (GE Healthcare), ÄKTA FPLC (GE Healthcare) and UNICORNTM 5.01 software. The protein was eluted with elution buffer [20 mM NaH2PO4.NaOH pH 7.5, 0.5 M NaCl, 0.5 M imidazole (Acros Organics), 10% glycerol]. The purification was conducted with the size-exclusion chromatography (SEC) using HiLoad 16/600 Superdex 200 Prep grade gel filtration column (GE Healthcare) and ÄKTA FPLC system (Fig. S3). To visualize the enzymatic activity and for comparison to phage-mediated lysis, a standard spot assay was used. Log-phase *Pseudomonas* strains were used to prepare bacterial lawn onto Trypticase Soy Agar plates (TSA, BioMérieux, France) using the double-agar overlay technique^71^. After drying, either 10 µl of serially diluted recombinant enzyme or 10 µl the phage suspension (10^6^ pfu/ml) was spotted onto the bacterial lawn. After overnight incubation at 37 °C, plates were observed for formation of clear zones (halo) or lytic zones (plaque) surrounded by a halo formed by the spotted LKA1gp49 recombinant protein and the LKA1 bacteriophage, respectively (Fig. S1A).

### Characterization of enzyme in solution

To check enzyme sensitivity to SDS, the sodium dodecyl sulfate-polyacrylamide gel electrophoresis (SDS-PAGE) was performed according to method described earlier^72^. The pH stability of LKA1gp49 at the concentration of 0.1 mg/ml was assessed after 1 h and 3 h of incubation using following buffers: pH 2 (hydrochloric acid/potassium), pH 4–8 (citric acid –disodium phosphate), pH 10 (sodium carbonate/sodium bicarbonate), and pH 12 (potassium chloride/ sodium hydroxide). For the temperature sensitivity tests, protein samples were incubated in PBS (pH 7.4) at 60 °C, 70 °C, 80 °C, 90 °C and 100 °C for 15 min and 60 min. After the incubation, LKA1gp49 was serially diluted and 10 µl of following concentrations: 100 µg/ml, 50 µg/ml; 10 µg/ml; 5 µg/ml; 1 µg/ml, 0.5 µg/ml and 0.1 µg/ml were spotted on PAO1 lawn (spot assay)^71,73^. The enzyme, suspended in PBS (pH 7.4) and incubated at RT, was used as a control.

### Phage and depolymerase specificity to bacterial receptor

The specificity of the LKA1gp49 recombinant protein and phage LKA1 for particular bacterial surface receptors was tested on PAO1 (serotype O5) and its mutants deficient in the biosynthesis of A-band and B-band O-polysaccharide, flagella, type IV pili, or alginate production (Table 1). The spot assay was used to determine bacterial susceptibility to phage-mediated lysis and enzyme degradation. A drop of the phage suspension (10^6^ pfu/ml) and 10 µl of serially diluted recombinant enzyme were spotted on a bacterial lawn and incubated at 37 °C. Plates were inspected after 4–6 h and again after 18 h for the presence of a lysis zone or halo zone^71,73^.

### Cloning and purification of gp49d and gp61d

Deletion mutants of LKA1gp49 and phi297 gp61 (called gp49d and gp61d, respectively) that did not contain the particle-binding domain (residues 1–167 for gp49 and residues 1–142 for gp61) were cloned to the expression vector pTSL (KU314761.1) using primers:

gp49dF: ATAGGATCCGTCGGTCAAAGCCTACAGTTT;

gp49dR: TATCTCGAGTCACACCTCATAAATACCTG;

gp61dF: ATAGGATCCACCGTAGCCGACCGCCTG;

gp61dR: ATACTCGAGTCAAATATCATCTAGGCTAGCA.

LKA1gp49 and phi297 gp61 were expressed as a downstream fusion to *E. coli* protein SlyD as described^74^. A Se-methionine (SeMet) derivative of gp61d was produced with the help of the SelenoMet kit (Molecular Dimensions, Suffolk, UK) according to the manufacturer’s protocol and was purified using the procedure established for the native protein.

### Crystallization, X-ray data collection and structure determination

The initial crystallization conditions for gp49d and gp61d were found using crystallization kits produced by Jena Bioscience. The initial sitting drop hits were scaled up and reproduced using the hanging drop technique. Gp49d was crystallized in 8% PEG 6000, 0.8 M NaCl, 0.1 M Na Acetate pH 3.5 at 18 °C and concentration of 20 mg/ml. Gp61d and its SeMet derivative was crystallized in 12% PEG 4000, 0.2 M ammonium acetate, 0.1 M HEPES pH 7.0 at 18 °C and concentration of 20 mg/ml. Crystallographic diffraction data were collected at the Swiss Light Source at the beamline PX-I (X06SA) in a shutter-less regime with a 360° rotation of the crystal split into 0.25° frames. The datasets were indexed and reduced with the help of XDS^75^. The structure of gp61d was solved by the Single wavelength Anomalous Diffraction technique with the help of a SeMet derivative and a dataset collected at the Se K absorption edge (Supplementary Table 3). The Se sites were found with the help of SHELXD and HKL2MAP programs^76,77^. The structure of gp49 was solved by the molecular replacement technique^78,79^ with the help of the PHASER program^80^ using gp61d as a search model. The initial atomic models were built by Buccaneer^81^, ARP/wARP^82^ and then refined with Phenix^83^ and Coot^84^ (Supplementary Table 3). The refined models were deposited to the Protein Data Bank under the accession numbers 4RU4 for gp49d and 4RU5 for gp61d.

### Isolation and cleavage of the OSA with LKA1gp49 depolymerase

*P. aeruginosa* strain 170005 belonging to IATS serotype O5 was kindly provided by B. Lányi (Institute of Hygiene, Budapest, Hungary). Cells were cultivated and LPS was isolated by the phenol-water method^85^ and degraded with 2% HOAc as described^51^ to give OSA. An OSA sample (10 mg) in 50 mM TrisHCl buffer pH 8.0 was treated with a solution of LKA1gp49 (1 mg/ml) for 16 h at 20 °C. The products were fractionated by gel-permeation chromatography sequentially on a column (56 × 2.6 cm) of Sephadex G-50 Superfine (Amersham Biosciences, Sweden) in 0.05 M pyridinium acetate buffer pH 4.5 and a column (90 × 2.5 cm) of TSK HW-40 (S) (Merck, Germany) in 1% HOAc monitored with a differential refractometer (Knauer, Germany) to give oligosaccharides **1** and **2** (7.5 and 0.9 mg, respectively).

### NMR spectroscopy and mass spectrometry

Samples were deuterium-exchanged by freeze-drying from 99.9% D_2_O and then examined as solutions in 99.95% D_2_O. NMR spectra were recorded on a Bruker Avance II 600 MHz spectrometer at 30 °C. Sodium 3-trimethylsilylpropanoate-2,2,3,3-d_4_ (δ_H_ 0, δ_C_ −1.6) was used as the internal reference for calibration. Two-dimensional NMR spectra were obtained using standard Bruker software, and Bruker TopSpin 2.1 program was used to acquire and process the NMR data. A mixing time of 100 and 150 ms was used in ^1^H,^1^H TOCSY and ^1^H,^1^H ROESY experiments, respectively. The ^1^H,^13^C HMBC spectrum was recorded with a 60-ms delay for evolution of long-range couplings. HR ESI MS was performed on a Bruker micrOTOF II instrument in the negative ion mode. A sample (~50 ng/µl) was dissolved in a 1:1 (v/v) water–acetonitrile mixture and injected with a syringe at flow rate 3 µl/min. Mass range was from *m*/*z* 50 to *m*/*z* 3000. Capillary entrance voltage was set to 3200 V. Nitrogen was applied as drying gas; interface temperature was set at 180 °C. Internal calibration was done with ESI Calibrant Solution (Agilent).

### LPS degradation assay for biology experiments

SDS-PAGE analysis using the Marolda protocol has been used to visualize the LPS degrading activity of the analyzed protein on the PAO1 host strain and its *rmd* (GDP-4-keto-6-deoxy-D-mannose reductase) knock out mutant. This mutant is deficient in A-band LPS synthesis, but produces smooth B-band LPS. Bacterial cultures of PAO1 and *Δrmd* strains were incubated with the recombinant LKA1gp49 depolymerase (0.05 mg/ml) for 1 h, 2 h and 6 hours. Afterwards, LPS was isolated by the phenol-water extraction, according to a modified version of the Marolda method^86^. The LPS samples were separated using the Tricin SDS-PAGE protocol^86^ and silver stained according to Tsai and Frasch^87^. The LPS from bacterial cultures incubated without enzyme was prepared as a control.

### Phage and depolymerase degradation of biofilm covering Nephrophane membrane

The ability of LKA1gp49 and *Pseudomonas* phage LKA1 to degrade the biofilm matrix was evaluated by laser interferometry method as described previously^56^. The Nephrophane (VEB Filmfabrik, Wolfen, Germany) a microporous, highly hydrophilic membrane made from cellulose acetate (trio-acetate cel-(OCO-CH_3_)_n_) was covered by a PAO1 biofilm grown for 72 h at 37 °C in TSB. The level of membrane coverage by biofilm was 92.4%. Next, the biofilm was treated for 4 hours at 37 °C with phage LKA1 (5 × 10^8^ pfu/ml) or recombinant depolymerase LKA1gp49, at a concentration of 2 and 0.02 mg/ml. The degradation of biofilm was assessed as the permeability increase of its matrix for low molecular compounds (TSB). The quantitative measurements of TSB diffusion through biofilm structure treated with phage and enzyme was tested by laser interferometry method exactly as presented elsewhere^55,56^.

### The antimicrobial activity of depolymerase/antibiotic composition

The minimum inhibitory concentration (MIC) of gentamicin sulfate (GE, MP Biomedicals, CA, USA) and ciprofloxacin (CIP, Fluka, Sigma-Aldrich) on PAO1 were determined by the broth microdilution technique according to Clinical and Laboratory Standards Institute (CLSI) recommendations (http://clsi.org/). The effect of CIP and GE at final concentrations of 0.018–1 µg/ml and 0.06–4 µg/ml, respectively, were checked separately and in the presence of 0.05 µg/ml of LKA1gp49. After incubation (20 h at 37 °C), the OD_600_ was checked using ASYS UVM 340 Microplate Reader (Biochrom Ltd, UK). The serial dilution and colony count of the culture were calculated for wells corresponding to ½ of the MIC. Negative controls consisted of MHB, MHB + antibiotic/enzyme at particular concentration. The positive controls included the PAO1 culture in MHB and PAO1 culture in MHB treated with depolymerase. No differences in OD_600_ were observed for both positive controls. The experiment was performed at least in triplicate.

### The efficiency uptake of phagocytic cells

The LKA1gp49 influence on the adhesion and PAO1 efficiency uptake was tested using the THP-1 human PBMC cell line (ATCC TIB-202). Cell lines were cultured with standard RPMI-1640 medium enriched with 10% fetal bovine serum, 1% GlutaMAX and Antibiotic-Antimycotic (media and supplements from Gibco, Thermo Fisher Scientific, Waltham, MA, USA). The THP-1 cell line was differentiated into macrophages by culturing with 100 ng/ml addition of phorbol-12-myristate-13-acetate (PMA, Sigma-Aldrich). The culture was maintained in 24-well polystyrene plates with a coverslip placed on the bottom (Thermanox Plastic NUNC Coverslips 13 mm). Each coverslip (covered with about 1.5 × 10^5^ of THP-1 derived macrophages) was placed for one hour in the well containing 1.5 × 10^7^ cfu of *P. aeruginosa* PAO1 (non-treated or pretreated with 50 µg/ml solution of LKA1gp49 for one hour). After one hour the coverslips were washed. The total number of bacteria (engulfed and adhered) was estimated by transferring coverslips into a 0.4% Triton X-100 solution for 15 minutes. Subsequently, the obtained bacterial suspension was serially diluted and plated. To estimate the number of bacteria engulfed by macrophages, coverslips were transferred to the 0.4 mg/ml GE solution for one hour. Next, the same coverslips were immersed in the 0.4% Triton X-100 solution for 15 minutes. Finally, the resulting bacterial suspension was serially diluted and plated. The number of bacteria adhered to the macrophage cell surface was quantified by subtraction of the number of engulfed bacteria from the total number of bacteria (engulfed and adhered).

### Serum complement activity

The assay was performed according to the method described elsewhere^88^. A log-phase bacterial culture was suspended in 50% sheep serum (SNS, ProAnimali, Wroclaw, Poland) or heat-inactivated sheep serum (SIS) for two hours at 37 °C. The numbers of viable bacteria were determined by serial dilution and plating (in triplicate) on TSA plates (BioMérieux, France), immediately following inoculation, and at half-hour intervals thereafter. The serum bactericidal effect was calculated as a percentage survival, using the bacterial counts when incubated with SIS values as 100%.

### *Galleria mellonella* larvae infection model

The influence of LKA1gp49 enzyme *P. aeruginosa* PAO1 virulence was tested using *Galleria mellonella* larvae model previously outlined by Cullen *et al*.^89^. After one week of acclimation, larvae selected in terms of size and appearance were inoculated (by injection into the hindmost proleg) with 10 cfu of *P. aeruginosa* PAO1. The experiment was divided into two variants, differing in the way of the enzyme administration. In the first setup, described by Olszak *et al*.^90^, the LKA1gp49 solution (5 or 50 µg/ml) was injected within 15 minutes after introduction of the bacterial cells to larvae hemocoel. In the second setup, larvae were injected with bacteria (10^5^ cfu/ml) pretreated (1 h) incubated with LKA1gp49 (50 µg/ml). Infected larvae were incubated at 37 °C for 72 hours and their viability was checked every 8 hours. Experiments were performed in triplicate (10 larvae per trial).The graphical presentation and statistical analysis of the results were performed using the GraphPad Prism software (GraphPad Software, Inc., La Jolla, USA). For the statistical analysis of the larvae survival curves, the log-rank Mantel-Cox test was used. *P*-values < 0.05 were considered as statistically significant.

### Ethical approval

This article does not contain any studies with human participants or animals performed by any of the authors.

## Electronic supplementary material

Supplementary materials
