# Supplementary material for: The O-specific polysaccharide lyase from the phage LKA1 tailspike reduces Pseudomonas virulence
Source: Sci Rep. 2017 Nov 24;7:16302. doi: 10.1038/s41598-017-16411-4 (PMC5701251; doi:10.1038/s41598-017-16411-4)
Supplement: Supplementary file 1 — Supplementary materials [file 41598_2017_16411_MOESM1_ESM.pdf]

The O-specific polysaccharide lyase from the phage LKA1 tailspike reduces *Pseudomonas* virulence

Tomasz Olszak[1], Mikhail M. Shneider[2,3], Agnieszka Latka[1], Barbara Maciejewska[1], Christopher Browning[4], Lada V. Sycheva[5], Anneleen Cornelissen[6], Katarzyna Danis-Wlodarczyk[1,6], Sofya N. Senchenkova[7], Alexander S. Shashkov[7], Grzegorz Gula[1], Michal Arabski[8], Slawomir Wasik[9], Konstantin A. Miroshnikov[2], Rob Lavigne[6], Petr G. Leiman[3], Yuriy A. Knirel[7] and Zuzanna Drulis-Kawa[1,*]

[1]Institute of Genetics and Microbiology, University of Wroclaw, Wroclaw, 51-148, Poland

[2]Shemyakin-Ovchinnikov Institute of Bioorganic Chemistry, Russian Academy of Sciences, Moscow, 117997, Russia

[3]University of Texas Medical Branch, Department of Biochemistry and Molecular Biology, Sealy Center for Structural Biology and Molecular Biophysics, Galveston, TX 77555-0647, USA

[4]Vertex Pharmaceuticals (Europe) Ltd, Abingdon, Oxfordshire OX14 4RW, UK.

[5]Affinivax Inc., Cambridge, 02139-3543 Massachusetts, USA

[6]Laboratory of Gene Technology, KU Leuven, Leuven, 3001, Belgium

[7]N. D. Zelinsky Institute of Organic Chemistry, Russian Academy of Sciences, Moscow, 119991, Russia

[8]Department of Biochemistry and Genetics, Institute of Biology, The Jan Kochanowski University in Kielce, Kielce, 25-406, Poland

[9]Department of Molecular Physics, Institute of Physics, The Jan Kochanowski University in Kielce, Kielce, 25-406, Poland

Correspondence:

Zuzanna Drulis-Kawa, e-mail: zuzanna.drulis-kawa@uwr.edu.pl

Supplementary Table 1. The stability of LKA1gp49 (spot test).

	**Enzyme concentration [µg/ml]**						
	**100**	**50**	**10**	**5**	**1**	**0.5**	**0.1**
**pH 2/1h**	-	-	-	-	-	-	-
**pH 2/3h**	-	-	-	-	-	-	-
**pH 4/1h**	+	+	+	+	-	-	-
**pH 4/3h**	+	+	+	+	-	-	-
**pH 6/1h**	+	+	+	+	+	-	-
**pH 6/3h**	+	+	+	+	+	-	-
**pH 7.4/1h**	+	+	+	+	+	+	-
**pH 7.4/3h**	+	+	+	+	+	+	-
**pH 8/1h**	+	+	+	+	+	+	-
**pH 8/3h**	+	+	+	+	+	+	-
**pH 10/1h**	+	+	+	+	+	+	-
**pH 10/3h**	+	+	+	+	+	+	-
**pH 12/1h**	+	+	+	+	+	-	-
**pH 12/3h**	+	+	+	+	+	-	-
**60°C/15 min**	+	+	+	+	+	+	-
**60°C/1h**	+	+	+	+	+	+	-
**70°C/15 min**	+	+	+	+	+	+	-
**70°C/1h**	+	+	+	+	+	+	-
**80°C/15 min**	+	+	+	+	+	+	-
**80°C/1h**	+	+	+	+	+	+	-
**90°C/15 min**	+	+	+	+	+	+	-
**90°C/1h**	+	+	+	+	+	-	-
**100°C/15 min**	+	+	+	+	-	-	-
**100°C/1h**	+	+	+	-	-	-	-

"+" visible opaque area; "-" lack of opaque area

Supplementary Table 2. ^{1}H and ^{13}C NMR data (δ, ppm; $^{3}J_{H,H}$, Hz) of oligosaccharide **1**.

**Residue**	**Monosaccharide**						**Acyl group at N2**		**Acyl group at N3**	
	C1<br>*H1*<br>$J_{1,2}$	C2<br>*H2*<br>$J_{2,3}$	C3<br>*H3*<br>$J_{3,4}$	C4<br>*H4*<br>$J_{4,5}$	C5<br>*H5*<br>$J_{5,6}$	C6<br>*H6*	C1	C2<br>*H2*	C1	C2<br>*H2*
**A**	98.1<br>*5.41*<br>2.3	48.7<br>*4.36*<br>6.2	42.4<br>*4.69*<br>5.8	107.0<br>*5.92*	145.3	169.7	175.0[a]	23.2[b]<br>*1.99*[c]	175.0[a]	23.3[b]<br>*2.01*[c]
**B**	100.7<br>*4.81*<br>4.1	49.6<br>*4.28*<br>11.2	74.8<br>*4.16*<br>3.5	71.7<br>*3.68*<br><1	68.2<br>*4.23*<br>6.5	16.5<br>*1.19*	175.2[a]	23.5[b]<br>*2.02*[c]		
**C**	71.4<br>*5.04*<br>3.1	45.7<br>*4.22*<br>11.8	52.5<br>*3.78*<br>n.d.	76.6<br>*4.06*<br>2.0	77.0<br>*4.52*	177.5	175.3[a]	23.1[b]<br>*2.04*[c]	163.4	19.9<br>*2.35*

^{1}H NMR chemical shifts are italicized. n.d., not determined.

[a-c] Assignment could be interchanged.

Supplementary Table 3. Crystallographic data statistics

**Crystal**	**Gp61d SeMet**	**Gp61d Native**	**Gp49d**
**Data collection**			
Space group	$C2_1$	$C2_1$	$P2_1$
Cell dimensions:			
a, b, c (Å)	210.993, 126.489, 83.411	209.648, 124.162, 83.189	113.771, 147.848, 117.325
α, β,γ (°)	90.000, 97.311, 90.000	90.000, 98.181, 90.000	90.000, 89.071, 90.000
Wavelength (Å)	0.97944	1.0	1.0
Resolution (Å)	50.0 – 2.00 (2.12 – 2.00)*	50.0 – 1.52 (1.61 – 1.52)	50.0 – 1.90 (2.02 – 1.90)
R_{meas} (%)	9.1 (76.0)	6.1 (31.8)	16.5 (60.4)
$CC_{1/2}$	99.8 (80.9)	99.8 (93.5)	98.9 (80.1)
I/σ_I	12.32 (2.27)	14.13 (3.62)	8.82 (2.74)
Completeness (%)	98.9 (95.5)	97.4 (93.1)	99.3 (98.0)
Redundancy	3.45 (3.36)	3.42 (3.30)	3.46 (3.46)
Anomalous signal#	1.24 (0.71)		
**Refinement**			
Software (version)		Refmac (5.7.0032)	Phenix (1.8.2_1309)
No. unique reflections		305,236	304,852
No. atoms			
Protein		14011	26,546
Ligand/ion		96	101
Water		3,133	5,661
Rwork / Rfree		0.079 / 0.120	0.129 / 0.174
B-factors			
Protein (Å^2)		15.39	11.96
Ligand/ion (Å^2)		36.24	26.49
Water (Å^2)		42.44	31.07
R.m.s. deviations			
Bond lengths (Å)		0.019	0.014
Bond angles (°)		1.784	1.133
Ramachandran plot			
Favored (%)		96.15	96.53
Allowed (%)		3.70	3.39
Outliers (%)		0.15	0.08

* The highest resolution shell is shown and the corresponding statistics are in parenthesis
# As calculated by the program XDS (77).

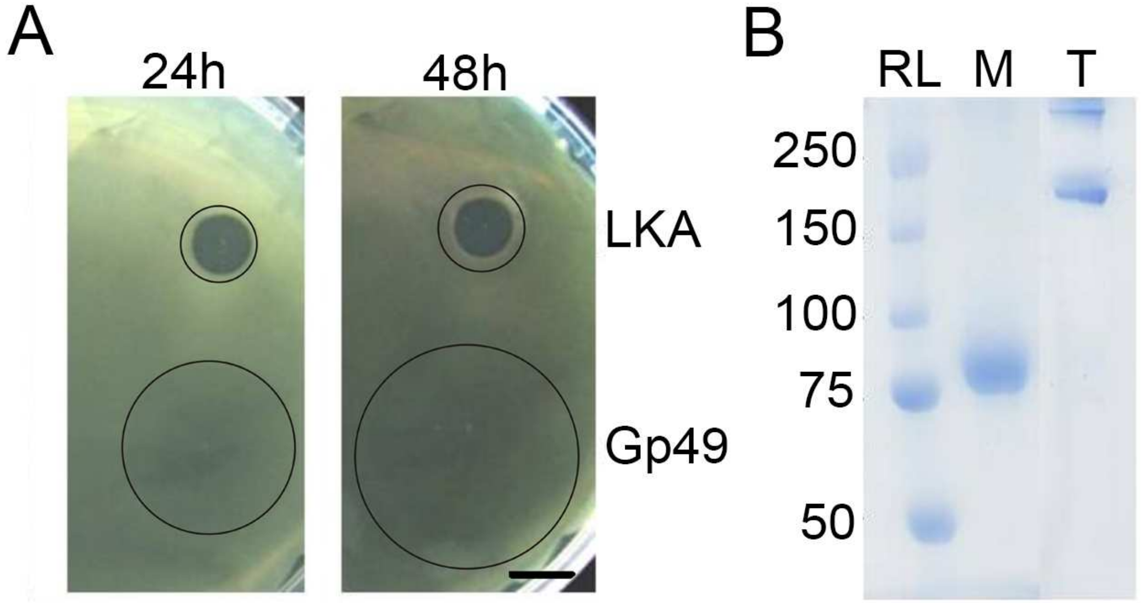

Figure S1. EPS-degrading activity (spot test) and trimerization of the LKA1gp49. (A) A 10 µl drop of 100 µg/ml of purified LKA1gp49 on a bacterial lawn of PAO1 creates after overnight incubation at 37°C an opaque area (below) identical to the halo zone formed around the lytic zone of phage infection (above). The diameter of this opaque-looking halo zone increases upon incubation. **(B)** Trimerization of LKA1gp49. Protein bands corresponding to the monomer (~80.0 kDA, M) and homotrimer (240.0 kDa, T) were observed on a 9% (w/v) SDS-PAGE gel with and without heating (5 min, 95°C) prior to electrophoresis, respectively. RL: Precision plus protein reference ladder (kDa; Bio-Rad).

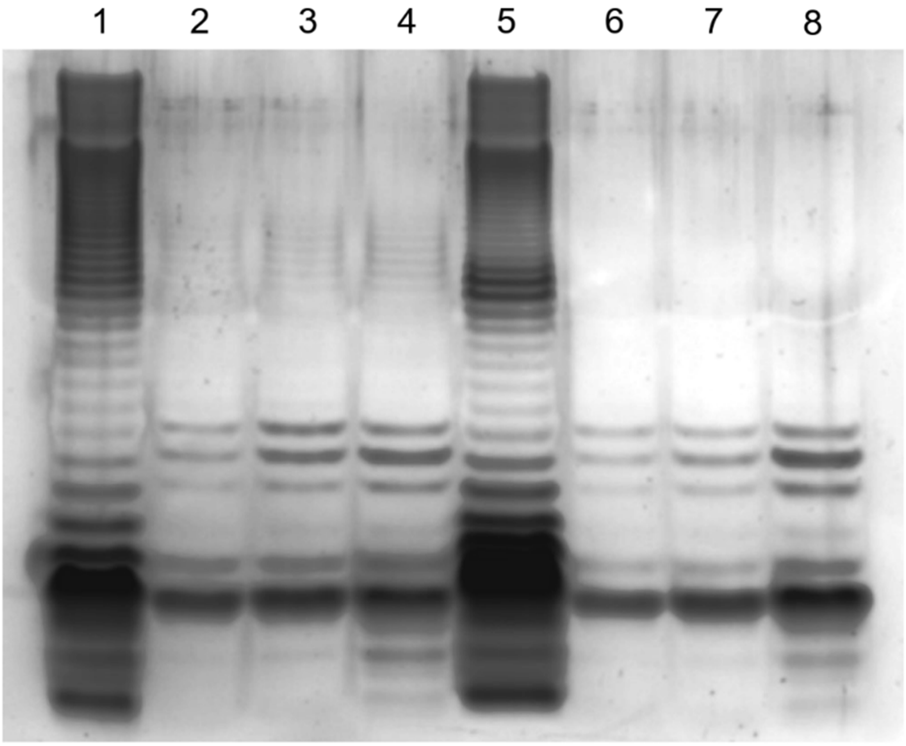

Figure S2. SDS PAGE analysis of PAO1 and *rmd* PAO1 mutant LPS degradation during LKA1gp49 treatment (50 µg/ml). 1 – PAO1 LPS, 2-4 – PAO1 LPS digested for 1h, 2h, 6h, respectively; 5 – *rmd* PAO1 LPS, 6-8 – *rmd* PAO1 LPS digested for 1h, 2h, 6h, respectively.

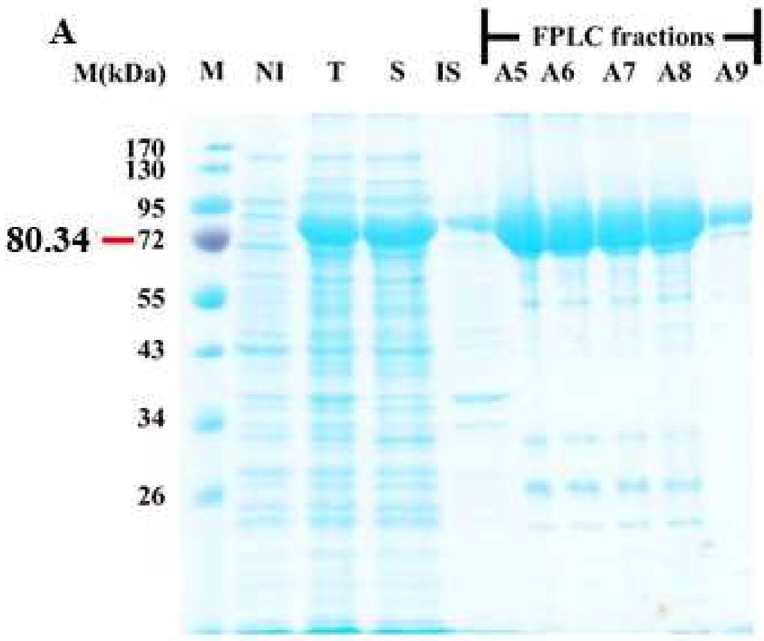

B

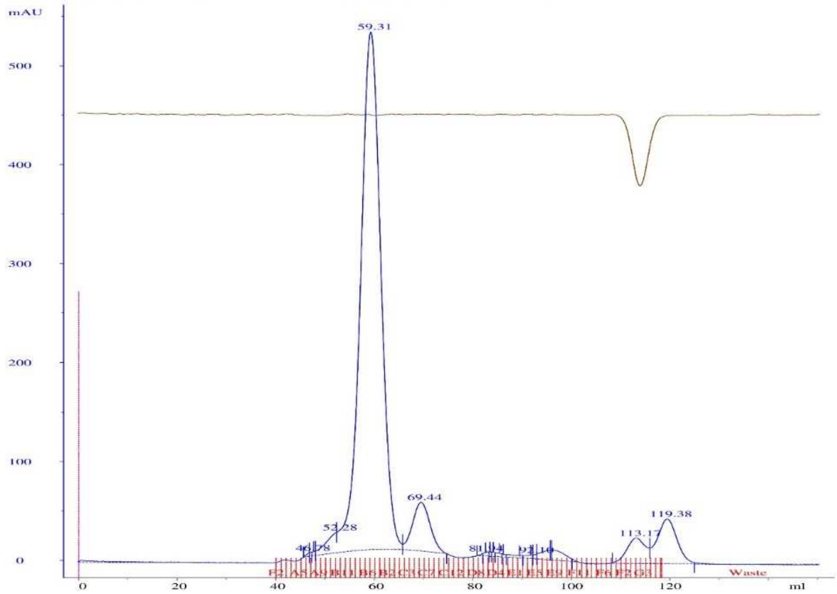

Figure S3. Expression and purification analysis of LKA1gp49. (A) 12% acrylamide SDS-PAGE gel with: PageRuler™ Prestained Marker (M); recombinant protein expression not induced (NI), total (T), soluble (S) and insoluble (IS) fractions; affinity chromatography fractions from FPLC purification, fractions A5-A9. Expected protein size (80.34 kDa) is marked on the left side of the gel with a red band. **(B)** Size exclusion chromatogram of elution fractions with peak after 59.31 ml of elution [y-axis =mAU, x-axis = eluted volume with fractions (ml)]. The SDS-PAGE gels are stained with GelCode™ Blue Safe Protein Stain (Thermo Scientific).
